# Supplementary material for: Neural mechanisms of credit assignment for delayed outcomes during contingent learning
Source: bioRxiv. 2024 Aug 8:2024.08.06.606895. Preprint. [Version 1] doi: 10.1101/2024.08.06.606895 (PMC11326259; doi:10.1101/2024.08.06.606895)
Supplement: Supplementary file 1 [file NIHPP2024.08.06.606895v1-supplement-1.pdf]

bioRxiv preprint doi: https://doi.org/10.1101/2024.08.06.606895; this version posted August 8, 2024. The copyright holder for this preprint (which was not certified by peer review) is the author/funder. This article is a US Government work. It is not subject to copyright under 17 USC 105 and is also made available for use under a CC0 license.

Supplemental Figures

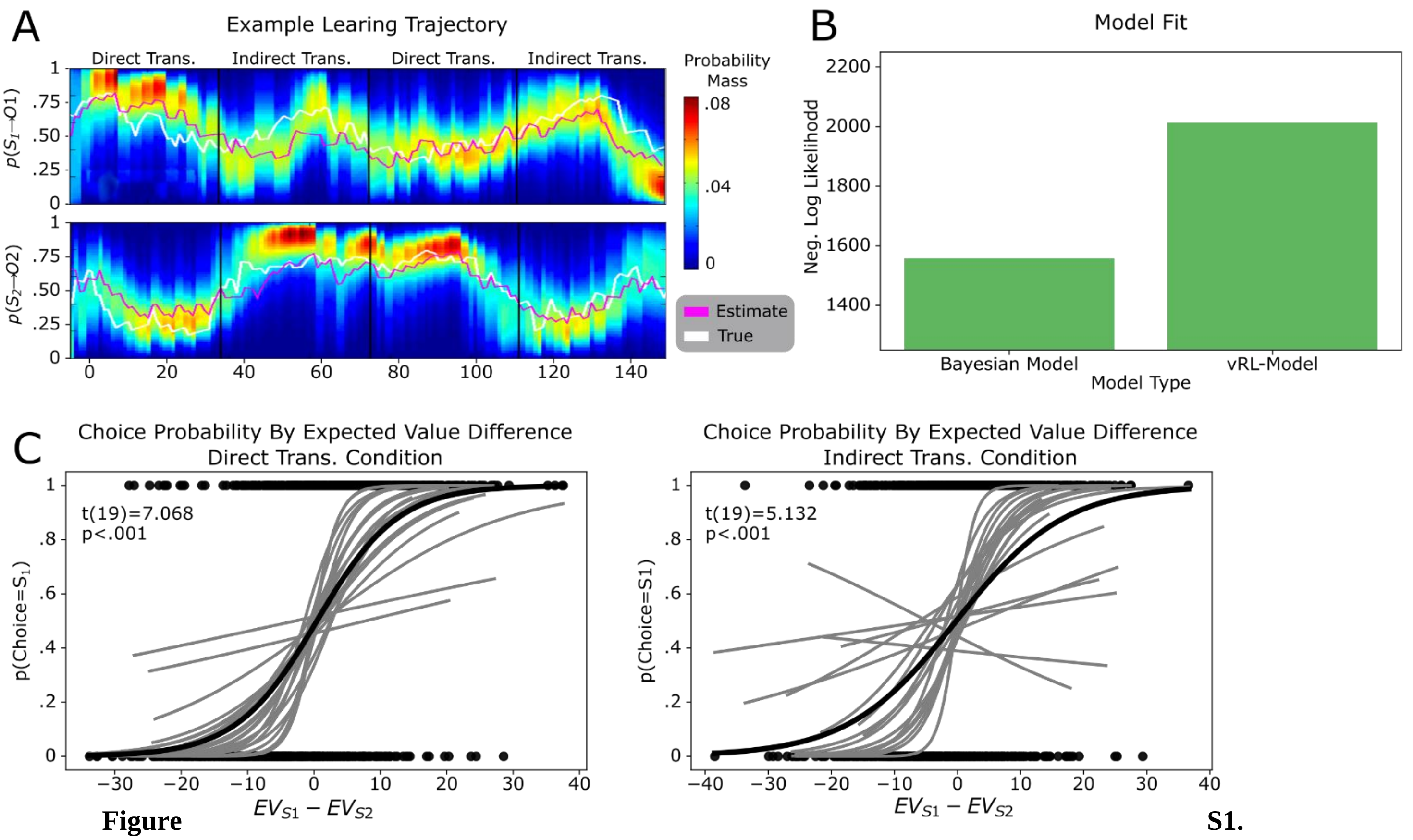

Figure S1. Follow up behavioral analyses

A. Example trajectory across the experiment of the belief estimates generated from the Bayesian learner. Top is the trajectory of S1, and the bottom is the trajectory of S2. While lines represent the true probability trajectory is shown in white and the estimated belief is shown in pink. Color heatmap shows the probability mass for each possible belief in S_x ->O1. B. Comparison of model fits between our Bayesian model and a value-based RL model (vRL) which used an interactive updating procedure to track the value of each shape based on the history of received rewards. The exceedance probability for the Bayesian model was 1, and 0 for the vRL model, suggesting that Bayesian model, which tracked transition probabilities between choices and outcomes, better fit participants actual choices compared to a value tracking model. **C**. Logistic regression curves estimating the change in choice probabilities given the expected value difference between choices. Gray line shows participant specific lines, and the black line shows the effect across groups (associated t-statistics are calculated across participants). The left side shows the effect in the direct transition condition and the right side shows the indirect transition condition.

bioRxiv preprint doi: https://doi.org/10.1101/2024.08.06.606895; this version posted August 8, 2024. The copyright holder for this preprint (which was not certified by peer review) is the author/funder. This article is a US Government work. It is not subject to copyright under 17 USC 105 and is also made available for use under a CC0 license.

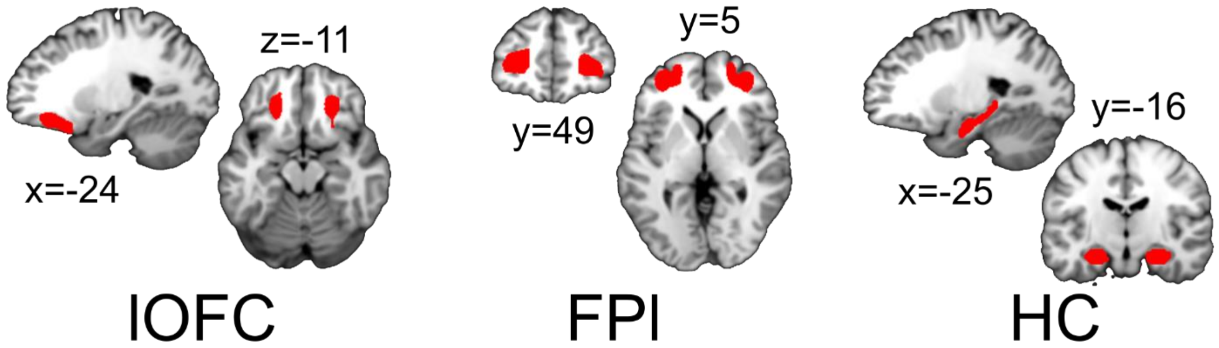

Figure S2. Pre-selected anatomical ROIs
Illustrations of pre-selected anatomical ROIs taken from Neubert et al, 2015. The lOFC ROI corresponds to index 9 and 30, FPl corresponds to indexes 14 and 35. The HC ROI was defined in Yushkevich et al., 2015.

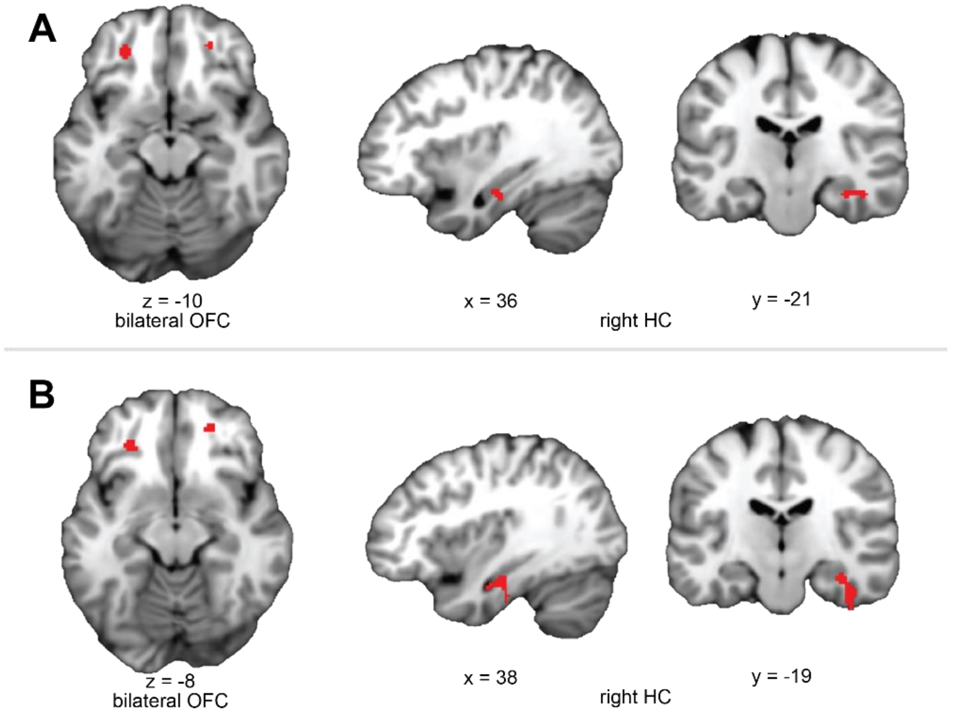

Figure S3. Functionally defined ROIs for in the direct transitions condition.
A) Despite having *a priori* defined anatomical ROIs for our decoding analysis of the causal choice, we wanted to test whether our results depended on these ROI definitions by using a data-driven approach. Here, we trained an SVM classifier to decode representations of the causal choice in run 1 of the direct transition condition, then tested the decoder on run 2 to find regions of the orbitofrontal cortex (OFC) and

bioRxiv preprint doi: https://doi.org/10.1101/2024.08.06.606895; this version posted August 8, 2024. The copyright holder for this preprint (which was not certified by peer review) is the author/funder. This article is a US Government work. It is not subject to copyright under 17 USC 105 and is also made available for use under a CC0 license.

hippocampus (HC) that significantly decoded causal choice representations at a significance level of $t(19) > 2.54$, $p < .01$, uncorrected. We then used these regions as ROIs for a separate analysis which trained the classifier in run 1 and tested the classifier in run 2. B) Shows ROIs generated from the same procedure as described in A, but the use of each run for training and testing are switched.

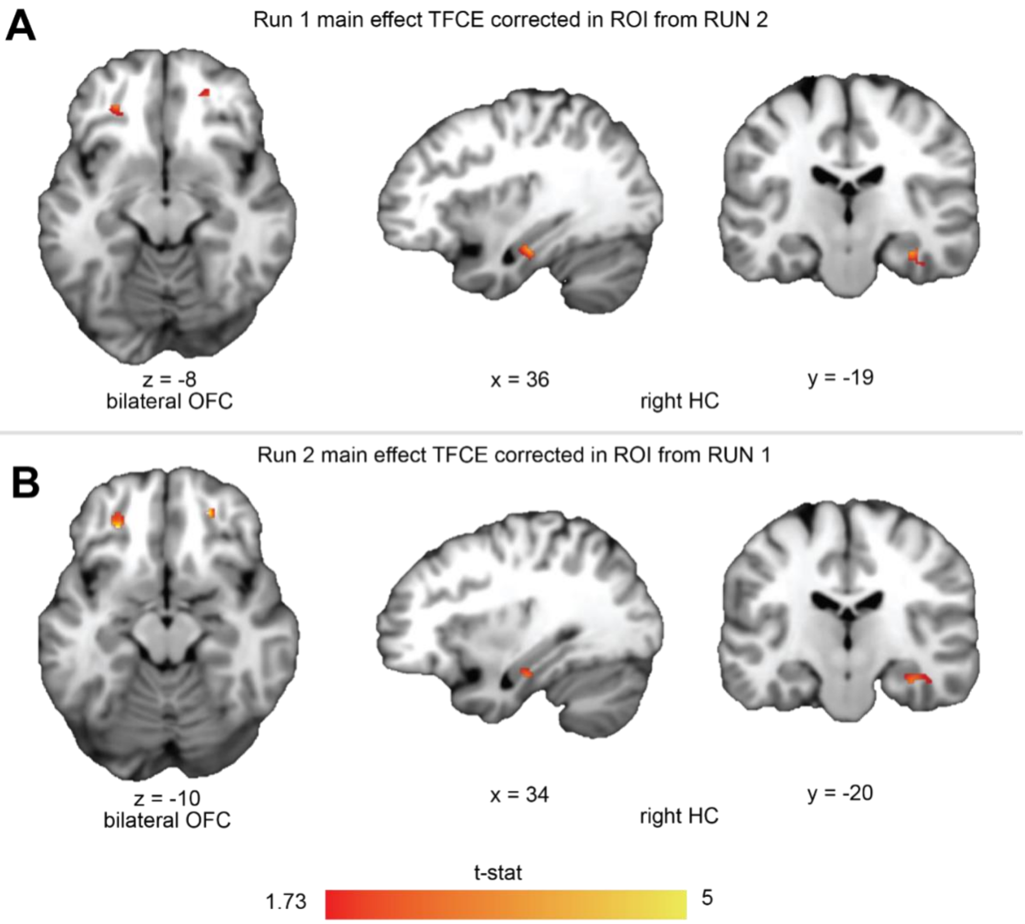

Figure S4. Main effect of choice decoding accuracy at the time of feedback TFCE corrected in each run of the direct transition condition

A. Regions of the OFC showing significant decoding of the causal choice in run 1 of the direct transition condition. Significance was tested using TFCE correction over voxels with the ROI generated from run 2, using the procedure described above (Fig.S1). For illustration, we show voxels that survive at threshold to $t(19)=1.73$, $p<.05$ uncorrected. B. Shows the same as A but for voxels in run 2, using the ROI generated from run 1.

bioRxiv preprint doi: https://doi.org/10.1101/2024.08.06.606895; this version posted August 8, 2024. The copyright holder for this preprint (which was not certified by peer review) is the author/funder. This article is a US Government work. It is not subject to copyright under 17 USC 105 and is also made available for use under a CC0 license.

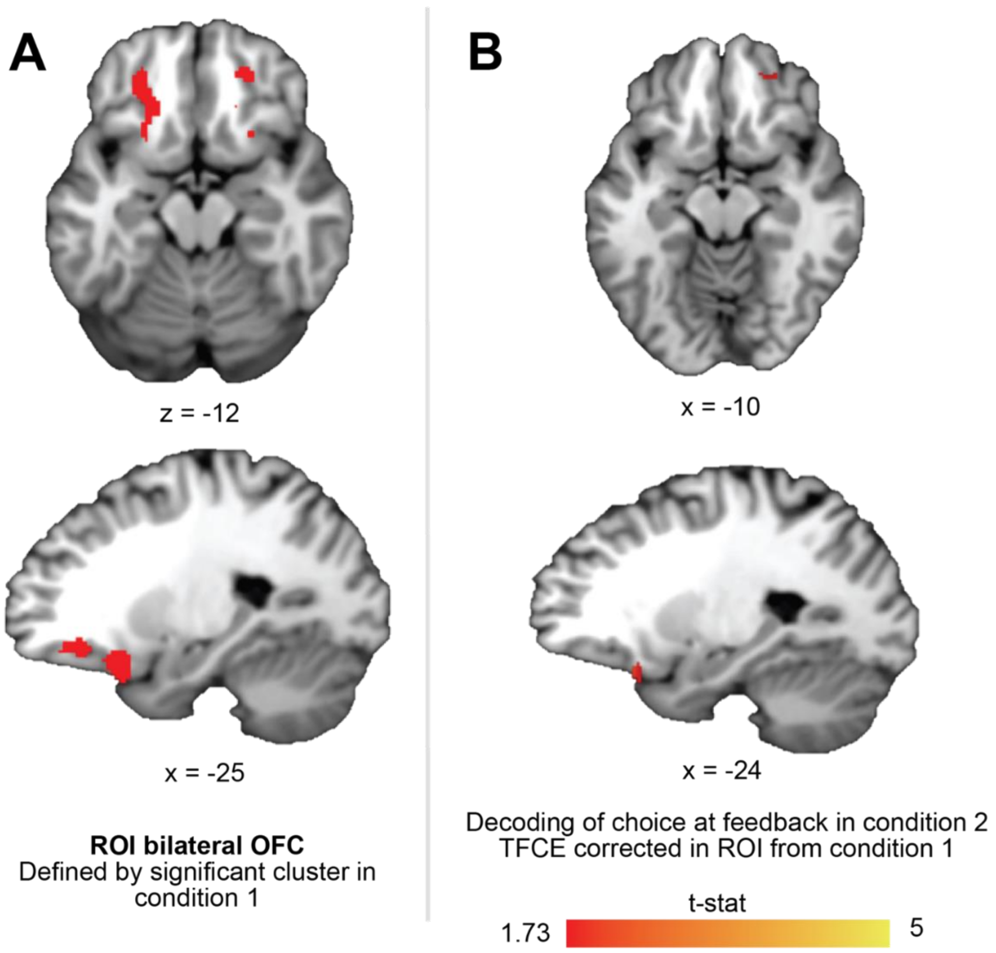

Figure S5. Significnant informaton connectivity between FPl and OFC in functionally defined ROI from direct transition condition

A. We did not observe signficiant decoding of the causal choice a in bilateral OFC ROI defined by significant cluseter in in the idirected transition condition. Thus, we used the accuracy map for decoding choices at feedback during the direct transition condition ($t(19) > 1.73$; $p < .05$) in the OFC, averaged across runs. B) We then used those cluster as ROI for TFCE correction for regions of the lOFC that showed significant information connectivity with FPl. We did this by testing for significant correlations between the trial-by-trial fidelity of pending representations in the FPl and causal choice representation during feedback in lOFC (see Methods).

bioRxiv preprint doi: https://doi.org/10.1101/2024.08.06.606895; this version posted August 8, 2024. The copyright holder for this preprint (which was not certified by peer review) is the author/funder. This article is a US Government work. It is not subject to copyright under 17 USC 105 and is also made available for use under a CC0 license.

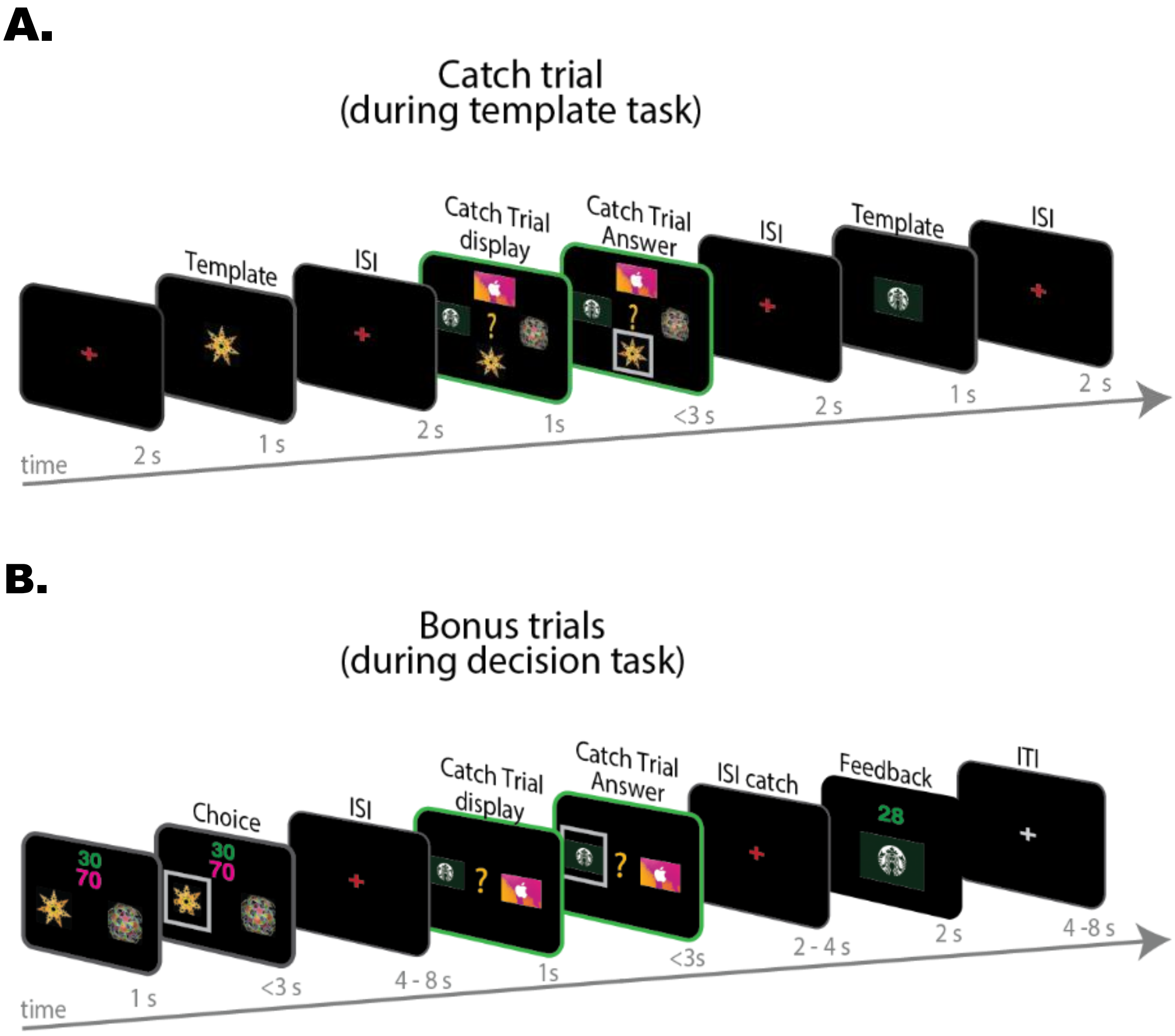

Figure S6. Depiction of catch trials

A. To ensure that participants where we included valuable *catch trials* in the passive observing "template task". Participants were asked to report which image out of the four (2 gift cards and 2 stimuli) was the last one presented on the screen. They were endowed an extra £10 from which we removed £1 for every incorrect response. There were four catch trials per template run. B. The decision task included "bonus trials" in which participants could predict which gift card they expected to see on the subsequent feedback screen given their choice. They were given 3£ extra on the final gift card that was given to them for every correct answer. The first run of the direct transition condition had two catch trials; the second run had one. Both runs of the indirect transition condition had one catch trial each.

bioRxiv preprint doi: https://doi.org/10.1101/2024.08.06.606895; this version posted August 8, 2024. The copyright holder for this preprint (which was not certified by peer review) is the author/funder. This article is a US Government work. It is not subject to copyright under 17 USC 105 and is also made available for use under a CC0 license.

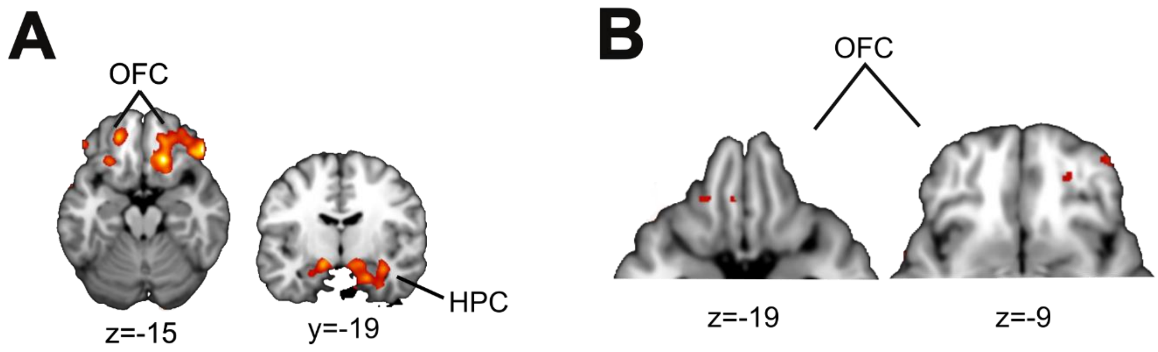

Figure S7. Control Analysis for Pending-to-Credit Assignment Information Connectivity in the Indirect Transition Condition

A. Axial (left) and coronal (right) slices through a t-statistic map showing the results of a control analysis in which test the proportion of correct classifications of causal stimulus information in OFC and HPC at the time of the outcome for trials in which the FPl showed correct classification for the causal stimulus during pending trials. The proportion of correct trials was compared to a permuted baseline of randomly drawn trials for each participant then combined over participants to create a t-statistic. B. Secondary control analysis in which we reran the classification analysis for causal choice stimulus information at the time of outcome, but only on trials where FPl was found to correctly decode pending causal choice information. Note that this test is different from A because we allowed the classifier to create a new hyperplane separating categories for only those trials in which the FPl decoding was "correct". For illustration, all maps are displayed at threshold of $t(19)=2.54$, $p<.01$ uncorrected. All effects survive small volume correction in a priori defined anatomical ROIs.
